# Does surgical intervention contribute to survival for patients with para‐aortic lymph node metastasis from colorectal cancer?

**DOI:** 10.1002/ags3.70019

**Published:** 2025-04-03

**Authors:** Akira Ouchi, Koji Komori, Sono Ito, Yusuke Kinugasa, Soichiro Ishihara, Akio Shiomi, Yukihide Kanemitsu, Takeshi Suto, Hiroki Takahashi, Yoichi Ajioka, the study group for PALNM projected by JSCCR, Akinobu Furutani, Akira Hirata, Atsushi Ishibe, Eiji Sunami, Heita Ozawa, Hideki Ueno, Hideyuki Ishida, Hiroyuki Egi, Ichiro Takemasa, Jun Watanabe, Junichiro Hiro, Kay Uehara, Kazushige Kawai, Koya Hida, Manabu Shiozawa, Masaaki Ito, Masakatsu Numata, Masanori Hotchi, Masaya Hiyoshi, Masayuki Ohue, Michio Itabashi, Ryo Inada, Shuntaro Nagai, Takaya Kobatake, Takeshi Kato, Tomohiro Yamaguchi, Yasumasa Takii, Yasumitsu Hirano, Yoshihiro Kakeji

**Affiliations:** ^1^ Department of Gastroenterological Surgery Aichi Cancer Center Hospital Nagoya Japan; ^2^ Department of Gastrointestinal Surgery Tokyo Medical and Dental University Tokyo Japan; ^3^ Department of Surgical Oncology The University of Tokyo Tokyo Japan; ^4^ Division of Colon and Rectal Surgery Shizuoka Cancer Center Hospital Nagaizumi‐cho Japan; ^5^ Department of Colorectal Surgery National Cancer Center Hospital Tokyo Japan; ^6^ Department of Gastroenterological Surgery Yamagata Prefectural Central Hospital Yamagata Japan; ^7^ Department of Gastroenterological Surgery Nagoya City University Graduate School of Medical Sciences Nagoya Japan; ^8^ Division of Molecular and Diagnostic Pathology, Graduate School of Medical and Dental Science Niigata University Niigata Japan

**Keywords:** colorectal cancer, overall survival, para‐aortic lymph node metastasis, relapse‐free survival, surgical resection

## Abstract

**Aims:**

There is a lack of compelling evidence supporting the benefit of surgical resection for para‐aortic lymph node metastasis (PALNM) from colorectal cancer (CRC). We aimed to investigate the true impact of surgical resection on survival for patients with PALNM from CRC.

**Patients and Methods:**

Patients diagnosed with PALNM from CRC at the Japanese Society for Cancer of the Colon and Rectum institutions between January 2011 and December 2015 were analyzed. Those who had received surgical resection and those who did not were matched one‐on‐one by the propensity score (PS)‐matching method. A total of 77 PS‐matched pairs extracted from 347 patients at 36 institutions were compared.

**Results:**

Thirty‐one (40.3%) patients each in the surgical resection and chemotherapy groups had distant metastasis other than PALNM, and the most dominant organ was the liver in 18 (23.4%) patients in both groups. In the surgical resection group, 56 (72.7%) patients achieved curative resection of all disease lesions, of which 49 (63.6%) were R0 resection. Three‐ and 5‐year relapse‐free survival of patients who achieved curative resection were 24.4% and 24.4%, respectively. Three‐ and 5‐year overall survival (OS) of patients in the surgical resection were 68.4% and 40.2%, which were significantly better than that in the chemotherapy groups (40.9% and 27.7%), respectively (Log‐rank *p* = 0.003).

**Conclusion:**

The OS of patients with surgical resection for PALNM was significantly better than those without surgical resection. These results highlight the benefit of surgical intervention to survival for patients with resectable PALNM.

## Graphical abstract

Overall survival of patients who underwent surgical resection was significantly better compared to that of patients who underwent chemotherapy without surgical resection. These results highlight the benefit of surgical intervention to survival for patients with isolated para‐aortic lymph node metastasis or with resectable liver metastasis.

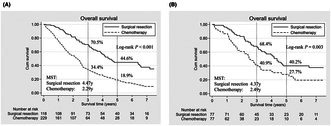

## 1 INTRODUCTION

Colorectal cancer (CRC) is the third most common cancer and second leading cause of cancer‐related deaths, with approximately 1.8 million new cases and about 881 000 new deaths worldwide.^1^ Up to 2% of CRC patients develop para‐aortic lymph node metastasis (PALNM), which is categorized as refractory distant lymph node metastasis and has a poor prognosis.^2^

Treatment options for metastatic CRC of the liver and/or lung include surgical metastasectomy and systemic chemotherapy, which, in combination, improve survival.^3^, ^4^ Meanwhile, there is no consensus on surgical metastasectomy for PALNM from CRC, given that it is generally considered a marker of underlying systemic disease. Although several studies have reported good prognoses for some patients with PALNM who underwent surgical resection,^2^, ^5^, ^6^, ^7^, ^8^, ^9^, ^10^, ^11^, ^12^, ^13^, ^14^, ^15^, ^16^, ^17^, ^18^ all of these were retrospective observational studies with a small sample population, possibly to have shown a false positive interpretation of the surgical resection results due to the selection bias. Therefore, compelling evidence supporting the benefit of surgical resection for PALNM is still lacking.

We have launched a study group for para‐aortic lymph node metastasis projected by JSCCR and reported the long‐term outcomes of patients who underwent PALNM resection as a multicenter retrospective study. The results of that study suggest the feasibility of PALNM resection and possible improvement of long‐term outcomes of patients with resectable PALNM.^19^ In the present study, we conducted a supplementary analysis of the JSCCR‐PALNM project to investigate the true impact of surgical resection on survival for patients with PALNM from CRC using a real‐world database of patients treated at dedicated institutions for CRC in Japan.

## 2 METHODS

### 2.1 Participants

A total of 463 patients diagnosed with PALNM from CRC at 36 institutions affiliated with JSCCR between January 2011 and December 2015 were registered in the JSCCR‐PALNM project, regardless of the type of treatment they underwent for PALNM. For patients who underwent surgical resection, those with clinically and histologically diagnosed PALNM were registered. Histological diagnosis was not mandatory for registration of patients who did not undergo surgical resection. Patients with a history of treatment for other pelvic‐abdominal malignancies and cases in which the primary tumor was the appendix were excluded prior to registration.

**FIGURE 1:**
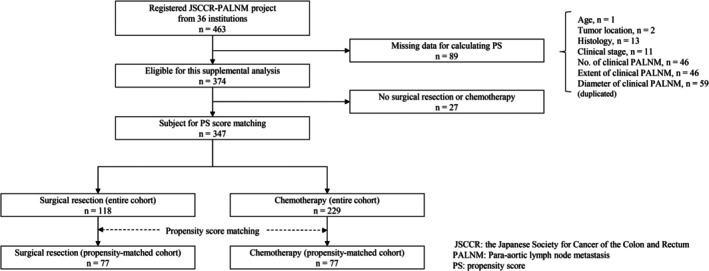
Flow diagram of patient selection. JSCCR, The Japanese Society for Cancer of the Colon and Rectum; PALNM, para‐aortic lymph node metastasis; PS, Propensity score.

After registration, 89 patients with missing propensity score (PS) data for this supplementary analysis were excluded as not eligible for analysis (Figure 1). Among the 374 eligible patients, 27 received no surgical resection or chemotherapy. Finally, 347 patients, including 118 patients who underwent surgical resection for PALNM (surgical resection group) and 229 patients who underwent chemotherapy without surgical resection (chemotherapy group), were included in the present supplementary analysis.

### 2.2 Treatment strategy for PALNM

The JSCCR guidelines recommend performing curative resection of the primary tumor and considering resection of the distant metastases when both the distant metastases and the primary tumor are resectable.^20^ These guidelines also recommend performing resection when recurrence is observed in a single organ and complete surgical resection of the recurrent tumor is possible, and even when recurrence is observed in more than a single organ, considering performing resection if recurrent tumors in all organs are resectable. Meanwhile, the actual indication for surgical intervention for patients with PALNM was argued by each institution according to the tumor spread, including cases involving concurrent distant metastasis.

With regard to preoperative chemotherapy, the JSCCR guidelines caution that there is lack of data on the efficacy and safety of preoperative chemotherapy for resectable recurrent lesions.^20^ Moreover, with regard to postoperative chemotherapy, these guidelines only recommend 5‐fluorouracil (FU) or tegafur and uracil/leucovorin after resection of liver metastasis. Accordingly, whether to perform preoperative or postoperative chemotherapy when treating PALNM is left entirely to the discretion of each institution.

### 2.3 Follow‐up and survival

The JSCCR guidelines recommend routine surveillance for recurrence after curative treatment of CRC.^20^ Surveillance for patients after curative resection of pathological Stage IV CRC entails physical examinations and the collection of laboratory data, including levels of tumor markers such as carcinoembryonic antigen (CEA) and carbohydrate antigen 19–9 (CA19‐9) every 3 months for the first 3 years and every 6 months for the subsequent 2 years, and CT scanning of the chest, abdomen, and pelvis every 6 months for 5 years.

Recurrence and survival rates for all patients were reviewed and augmented by a secondary chart review at each institution until any event or March 2021. For patients who underwent surgical resection, survival time was defined as the time from PALNM resection to any event. For those who did not undergo surgical resection, survival time was defined as the time from PALNM diagnosis to any event. Relapse‐free survival (RFS) was assessed for patients who achieved curative (R0/1) resection and was calculated as the time to any recurrence or death from any cause. Overall survival (OS) was calculated as the time to death from any cause.

### 2.4 Statistical analysis

Tumor staging and surgical radicality were recorded according to the JSCCR classification.^21^ The location of PALNM was recorded according to the Japanese Society of Clinical Oncology classification in the primary analysis^22^ and converted to two groups for this supplementary analysis: localized below renal vein (RV) and spread above RV.

Patient characteristics greatly differed between the 118 patients in the surgical resection group and the 229 patients in the chemotherapy group, since treatment selection for PALNM depends on tumor spread, in particular, when there is concurrent distant metastasis. Therefore, PS matching was used to adjust for baseline characteristics. PSs were estimated using age, sex, tumor location, histology, cT and cN classification, time to metastasis, the imaging finding of clinical PALNM, including the number, extent (spread above RV), and diameter, and concurrent distant metastasis, including to the liver, lung, distant lymph node, peritoneum, and bone metastasis, as covariates in multivariable logistic regression analysis. Patients were matched one‐on‐one using the estimated scores with a caliper of 0.03. C statistics for PSs estimated in the present study were adequate (area under the ROC curve = 0.8328), and the distribution of PSs sufficiently overlapped by treatment. After matching, 77 PS‐matched pairs were extracted from the surgical resection and chemotherapy groups, and the balance of selected variables was well adjusted.

Categorical variables were analyzed using Pearson's χ^2^ test. Continuous variables are presented as medians with interquartile ranges (IQRs) and were analyzed using the Mann–Whitney U test. Cumulative risks of RFS and OS were estimated by Kaplan–Meier survival analysis and compared using the log‐rank test. The hazard ratio (HR) and its 95% confidence interval (CI) were calculated by univariable Cox proportional hazard models. *p* < 0.05 was considered statistically significant. All tests were two‐sided, and α was set at 0.05. STATA 18.0 (StataCorp LLC, College Station, Texas, USA) was used for all statistical analyses.

## 3 RESULTS

### 3.1 Patient characteristics

Patient characteristics for the entire cohort and PS‐matched cohort are shown in Table 1. In the entire cohort, significant differences were observed in age, cT classification of the primary tumor, time to metastasis, number and extent of clinical PALNM, and concurrent distant metastasis between the surgical resection and chemotherapy groups.

**TABLE 1:** Patient characteristics in the entire cohort (*n* = 347) and propensity score‐matched cohort (*n* = 154).

	Entire cohort			Propensity score‐matched cohort		
	Surgical resection (*n* = 118)	Chemotherapy (*n* = 229)	*p* Value	Surgical resection (*n* = 77)	Chemotherapy (*n* = 77)	*p* Value
Age, years						
Median (IQR)	63 (54–69)	65 (59–72)	0.029	64 (56–72)	66 (60–72)	0.399
<65 yo	64 (54.2)	102 (44.5)	0.087	39 (50.7)	33 (42.9)	0.333
≥65 yo	54 (45.8)	127 (55.5)		38 (49.4)	44 (57.1)	
Sex, *n* (%)						
Female	49 (41.5)	100 (43.7)	0.702	30 (39.0)	32 (41.6)	0.742
Male	69 (58.5)	129 (56.3)		47 (61.0)	45 (58.4)	
Primary tumor location, *n* (%)						
Right colon	15 (12.7)	53 (23.1)	0.064	9 (11.7)	14 (18.2)	0.492
Left colon	57 (48.3)	101 (44.1)		40 (52.0)	35 (45.5)	
Rectum	46 (39.0)	75 (32.8)		28 (36.4)	28 (36.4)	
Histology, *n* (%)						
pap/tub	101 (85.6)	191 (83.4)	0.429	63 (81.8)	65 (84.4)	0.685
por/muc/sig	16 (13.6)	31 (13.5)		13 (16.9)	10 (13.0)	
other	1 (0.9)	7 (3.1)		1 (1.3)	2 (2.6)	
cT classification* of primary tumor, *n* (%)						
cT1	2 (1.7)	5 (2.2)	0.001	0	3 (3.9)	0.248
cT2	2 (1.7)	8 (3.5)		1 (1.3)	2 (2.6)	
cT3	72 (61.0)	88 (38.4)		40 (52.0)	33 (42.9)	
cT4	42 (35.6)	128 (55.9)		36 (46.8)	39 (50.7)	
cN classification* of primary tumor, *n* (%)						
cN0	12 (10.2)	27 (11.8)	0.207	8 (10.4)	7 (9.1)	0.472
cN1	25 (21.2)	68 (29.7)		20 (26.0)	18 (23.4)	
cN2	38 (32.2)	72 (31.4)		29 (37.7)	23 (29.9)	
cN3	43 (36.4)	62 (27.1)		20 (26.0)	29 (37.7)	
Time to metastasis, *n* (%)						
Synchronous	88 (74.6)	127 (55.5)	0.001	50 (64.9)	51 (66.2)	0.865
Metachronous	30 (25.4)	102 (44.5)		27 (35.1)	26 (33.8)	
Number of clinical PALNM						
Median (IQR)	1 (1–4)	2 (1–5)	<0.001	2 (1–4)	2 (1–4)	0.370
Solitary	60 (50.9)	77 (33.6)	0.002	38 (49.4)	31 (40.3)	0.257
Multiple	58 (49.2)	152 (66.4)		39 (50.7)	46 (59.7)	
Extent of clinical PALNM, *n* (%)						
Localized below RV	109 (92.4)	154 (67.3)	<0.001	69 (89.6)	67 (87.0)	0.616
Spread above RV	9 (7.6)	75 (32.8)		8 (10.4)	10 (13.0)	
Maximum diameter of clinical PALNM, mm						
Median (IQR)	9 (6–12)	9 (7–12)	0.367	9 (7–12)	9 (6–12)	0.576
10 mm	63 (53.4)	119 (52.0)	0.801	39 (50.7)	40 (52.0)	0.872
≥10 mm	55 (46.6)	110 (48.0)		38 (49.4)	37 (48.1)	
Concurrent distant metastasis**, *n* (%)						
Present	32 (27.1)	161 (70.3)	<0.001	31 (40.3)	31 (40.3)	1.000
Liver	18 (15.3)	91 (39.7)		18 (23.4)	18 (23.4)	
Lung	5 (4.2)	65 (28.4)		5 (6.5)	4 (5.2)	
Distant lymph node	6 (5.1)	63 (27.5)		6 (7.8)	6 (7.8)	
Peritoneum	6 (5.1)	22 (9.6)		5 (6.5)	5 (6.5)	
Bone	0	15 (6.6)		0	0	
Other	4 (3.4)	17 (7.4)		4 (5.2)	6 (7.8)	
No. of organs, *n* (%)						
Median (IQR)	0 (0–1)	1 (0–2)	<0.001	0 (0–1)	0 (0–1)	0.988
0	85 (72.0)	68 (29.7)	<0.001	45 (58.4)	46 (59.7)	0.786
1	27 (22.9)	79 (34.5)		26 (33.8)	23 (29.9)	
≥2	6 (5.1)	82 (35.8)		6 (7.8)	8 (10.4)	

Abbreviations: IQR, interquartile range; JSCCR, Japanese Society for Cancer of the Colon and Rectum; PALNM, para‐aortic lymph node metastasis; RV, renal vein.

* According to JSCCR classification.

** Duplicated.

In the PS‐matched cohort, 50 (64.6%) and 51 (66.2%) patients in the surgical resection and chemotherapy groups, respectively, had synchronous PALNM; 38 (49.4%) and 31 (41.3%) patients, respectively, had multiple PALNM, and eight (10.4%) and 10 (13.0%) patients, respectively, had lymph node metastasis spread above RV. With regard to concurrent distant metastasis, 31 (40.3%) patients each in the surgical resection and chemotherapy groups had a distant metastasis other than PALNM, with the most dominant organ being the liver in both groups (18 patients each; 23.4%). Six (7.8%) and eight (10.4%) patients in the surgical resection and chemotherapy groups, respectively, had more than two distant metastases other than PALNM.

### 3.2 Perioperative treatment and surgical details for surgical resection

Perioperative treatment and surgical details of patients who underwent surgical resection in the entire cohort and PS‐matched cohort are shown in Table 2. In the PS‐matched cohort, 14 (18.2%) patients received preoperative chemotherapy, including 12 (15.6%) with molecular‐targeted drugs. Forty‐three (55.8%) and 34 (44.2%) patients underwent systemic PALN dissection and lymph node excision, respectively, and the median (IQR) number of dissected PALN and pathologically confirmed PALNM were five (2 – 12) and two (1 – 4), respectively. Fifty‐six (72.7%) patients underwent concomitant resection of other organs, and the dominant procedure was primary tumor resection in 48 (62.3%), followed by hepatectomy in eight (10.4%).

**TABLE 2:** Perioperative treatment and surgical details of patients with surgical resection in the entire cohort (*n* = 118) and propensity score‐matched cohort (*n* = 77).

	Entire cohort	Propensity score‐matched cohort
	Surgical resection (*n* = 118)	Surgical resection (*n* = 77)
Preoperative chemotherapy, *n* (%)		
Present	23 (19.5)	14 (18.2)
Cytotoxic agent	23 (19.5)	14 (18.2)
FU monotherapy	0	0
L‐OHP doublet	20 (17.0)	12 (15.6)
CPT‐11 doublet	1 (0.9)	0
L‐OHP/CPT‐11 triplet	2 (1.7)	2 (2.6)
Molecular‐targeted drug	19 (16.1)	12 (15.6)
VEGF	13 (11.0)	8 (10.4)
EGFR	6 (5.1)	4 (5.2)
Concomitant radiotherapy	1 (0.9)	0
Surgical procedure for PALNM, *n* (%)		
Systematic PALN dissection	70 (59.3)	43 (55.8)
Lymph node excision	48 (40.7)	34 (44.2)
Surgical approach for PALNM, *n* (%)		
Laparotomy	93 (78.8)	66 (85.7)
Laparoscopy (including robot‐assisted)	25 (21.2)	11 (14.3)
Number of dissected PALN		
Median (IQR)	5 (2–12)	5 (2–12)
Number of pathological PALNM		
Median (IQR)	2 (1–4)	2 (1–4)
Concomitant resection**, *n* (%)		
Present	91 (77.1)	56 (72.7)
Primary tumor	82 (69.5)	48 (62.3)
Liver	8 (6.8)	8 (10.4)
Other	10 (8.5)	9 (11.7)
Postoperative complication, *n* (%)		
Clavien–Dindo grade IIIa or more	15 (12.7)	11 (14.3)
Anastomotic leakage	5 (4.**2)	4 (5.2)
SSI	3 (2.5)	2 (2.6)
Ileus	2 (1.7)	1 (1.3)
Lymphorrhea	0	0
Other	6 (5.1)	5 (6.5)
Curability		
Curative resection	93 (78.8)	56 (72.7)
R0	86 (72.9)	49 (63.6)
R1	7 (5.9)	7 (9.1)
Palliative resection (R2)	25 (21.2)	21 (27.3)
Postoperative chemotherapy, *n* (%)		
Present	84 (71.2)	57 (74.0)
Cytotoxic agent	82 (69.5)	56 (72.7)
FU monotherapy	14 (11.9)	10 (13.0)
L‐OHP doublet	58 (49.2)	41 (53.3)
CPT‐11 doublet	7 (5.9)	4 (5.2)
L‐OHP/CPT‐11 triplet	3 (2.5)	1 (1.3)
Molecular‐targeted drug	19 (16.1)	11 (14.3)
VEGF	15 (12.7)	9 (11.7)
EGFR	4 (3.4)	2 (2.6)

Abbreviations: CPT‐11, irinotecan; EGFR, epidermal growth factor receptor; FU, fluorouracil; IQR, interquartile range; L‐OHP, oxaliplatin; PALNM, para‐aortic lymph node metastasis; VEGF, vascular endothelial growth factor.

^**^ Duplicated

Fifty‐six (72.7%) patients achieved curative resection of all disease lesions, including PALNM, of which 49 (63.6%) were R0 resections. Fifty‐seven (74.0%) patients received postoperative chemotherapy, including 11 (14.3%) with molecular‐targeted drugs.

### 3.3 Primary tumor resection and first‐line regimen for chemotherapy

Primary tumor resection and first‐line regimen of patients who underwent chemotherapy in the entire cohort and PS‐matched cohort are shown in Table 3. In the PS‐matched cohort, 54 (70.1%) patients underwent primary tumor resection. All 77 patients had systemic chemotherapy, and many patients received oxaliplatin doublet (30; 39.0%) and a VEGF inhibitor (17; 22.1%). Detailed regimen data were missing in 32 (41.6%) patients for cytotoxic agents and 45 (58.4%) patients for molecular‐targeted drugs.

**TABLE 3:** Primary tumor resection and first‐line regimen of patients with chemotherapy in the entire cohort (*n* = 229) and propensity score‐matched cohort (*n* = 77).

	Entire cohort	Propensity score‐matched cohort
	Chemotherapy (*n* = 229)	Chemotherapy (*n* = 77)
Primary tumor resection, *n* (%)		
Absent	46 (20.1)	23 (29.9)
Present	183 (79.9)	54 (70.1)
First‐line chemotherapy, *n* (%)		
Present	229 (100)	77 (100)
Cytotoxic agent		
FU monotherapy	39 (17.0)	7 (9.1)
L‐OHP doublet	91 (39.7)	30 (39.0)
CPT‐11 doublet	6 (2.6)	2 (2.6)
L‐OHP/CPT‐11 triplet	15 (6.6)	6 (7.8)
Other	3 (1.3)	0
Unknown	74 (32.3)	32 (41.6)
Molecular‐targeted drug		
VEGF	56 (24.5)	17 (22.1)
EGFR	13 (5.7)	4 (5.2)
Unknown	122 (53.3)	45 (58.4)
Concomitant radiotherapy	3 (1.3)	1 (1.3)

Abbreviations: CPT‐11, irinotecan; EGFR, epidermal growth factor receptor; FU, fluorouracil; L‐OHP, oxaliplatin; VEGF, vascular endothelial growth factor.

### 3.4 Recurrence and survival

**FIGURE 2:**
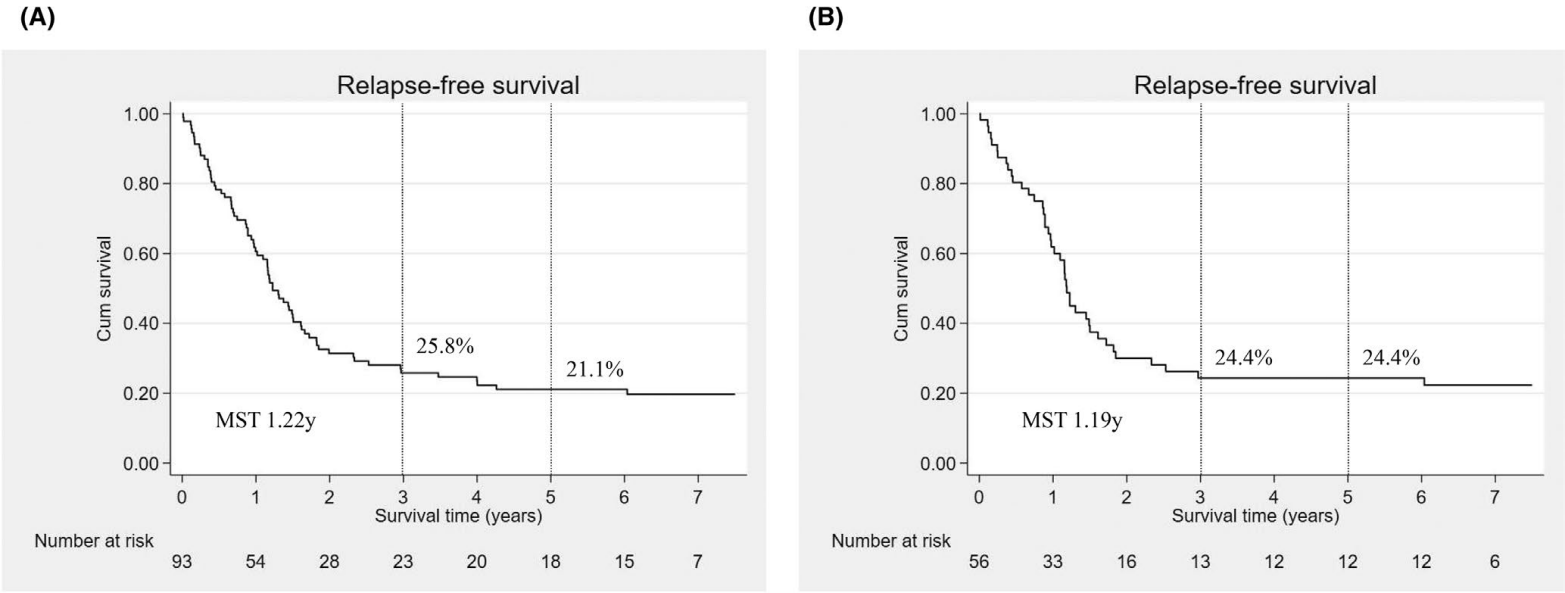
Relapse‐free survival of patients who achieved curative resection in the (A) entire cohort (*n* = 93) and (B) propensity score‐matched cohort (*n* = 56). MST, median survival time.

Median follow‐up periods of patients with curative resection for recurrence in the entire cohort and PS‐matched cohort were 1.19 (0.66–2.97) and 1.17 (0.77–2.75) years, respectively. In the PS‐matched cohort, 42 (75.0%) of 56 patients with R0/1 resection developed recurrence, with the most dominant organ being the lung (19; 33.9%) followed by lymph node (18; 32.1%) (Table 4). With regard to lymph node recurrence, PALN recurrence (13; 23.2%) was observed more than other distant lymph node recurrences (9; 16.1%). Three‐ and 5‐year RFS of patients who achieved curative resection were 24.4% and 24.4%, respectively, with a median survival time (MST) of 1.19 years (Figure 2).

**TABLE 4:** Recurrent sites of patients who achieved curative resection in the entire cohort (*n* = 98) and propensity score‐matched cohort (*n* = 56).

	Entire cohort	Propensity score‐matched cohort
	Curative resection (*n* = 98)	Curative resection (*n* = 56)
Recurrence^a^, *n* (%)	72 (77.4)	42 (75.0)
Lymph node	35 (37.6)	18 (32.1)
PALN	23 (24.7)	13 (23.2)
Other distant lymph node	20 (21.5)	9 (16.1)
Lung	31 (33.3)	19 (33.9)
Liver	18 (19.4)	13 (23.2)
Peritoneum	11 (11.8)	11 (19.6)
Bone	3 (3.2)	2 (3.6)
Other	7 (7.5)	6 (10.7)

Abbreviation: PALN, para‐aortic lymph node.

^a^ Duplicated.

**FIGURE 3:**
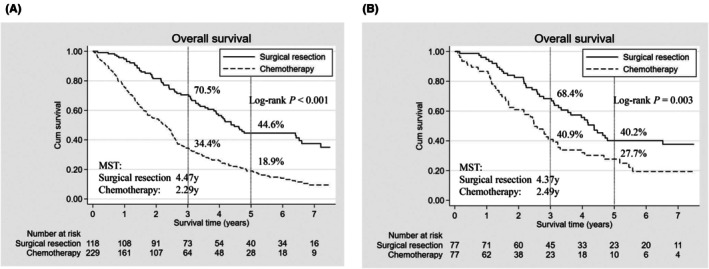
Overall survival of patients who underwent surgical resection and chemotherapy in the (A) entire cohort (*n* = 347) and (B) propensity score‐matched cohort (*n* = 154). MST, median survival time.

Median follow‐up periods for survival in the entire cohort and PS‐matched cohort were 3.75 (2.17–6.26) and 3.30 (2.17–6.18) years, respectively, in the surgical resection group, and 1.80 (0.80–3.30) and 1.97 (1.12–3.30) years, respectively, in the chemotherapy group. In the PS‐matched cohort, 3‐ and 5‐year OS of patients in the chemotherapy group were 40.9% and 27.7%, respectively, with an MST of 2.49 years (Figure 3). Meanwhile, 3‐ and 5‐year OS of patients in the surgical resection group were 68.4% and 40.2%, respectively, with a MST of 4.37 years, which were significantly better compared to those in the chemotherapy group (Log‐rank *p* = 0.003).

### 3.5 Subgroup analysis for OS in the PS‐matched cohort

**FIGURE 4:**
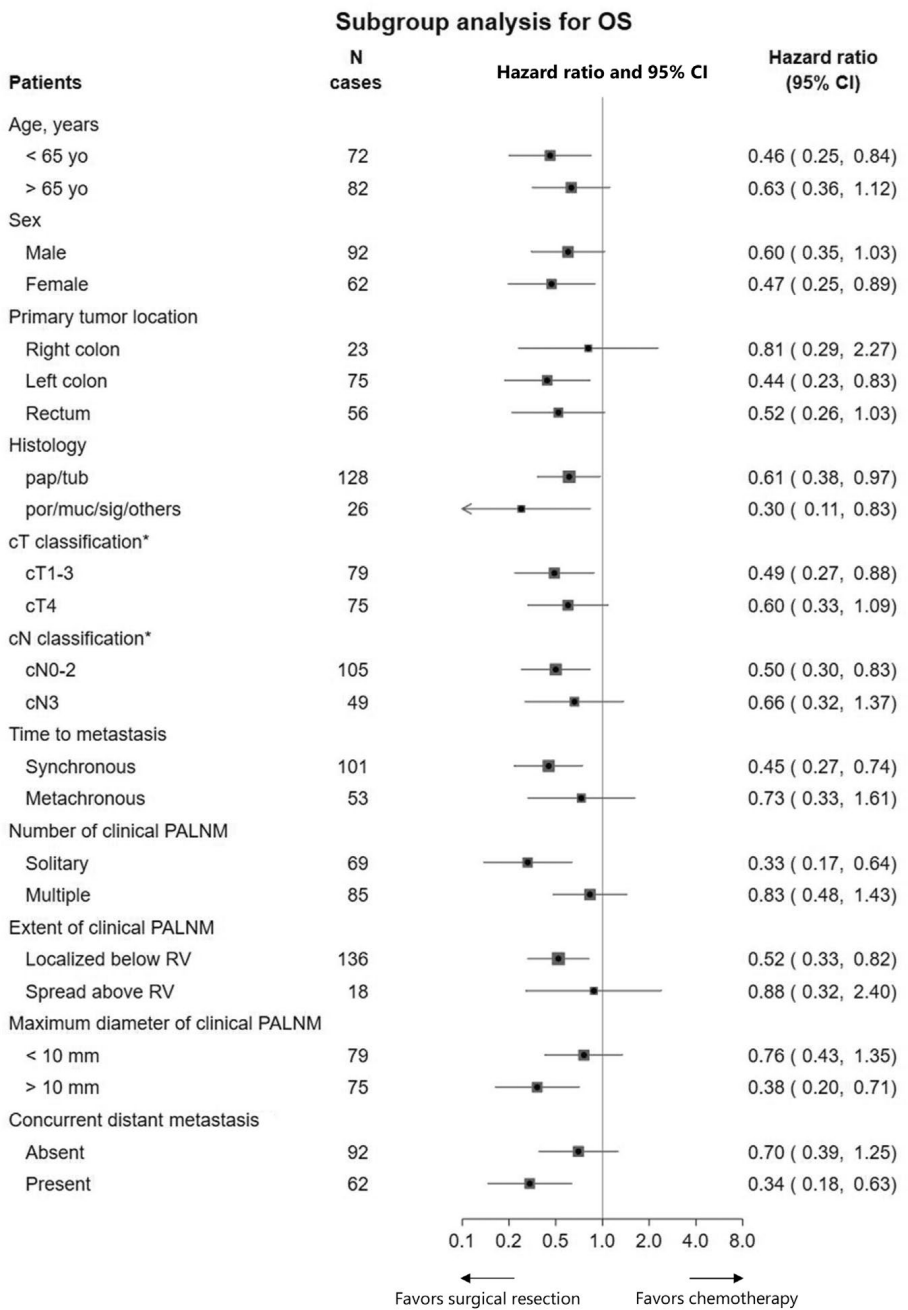
Subgroup analysis for overall survival in the propensity score‐matched cohort. CI, Confidence interval; JSCCR, Japanese Society for Cancer of the Colon and Rectum; OS, Overall survival; PALNM, Para‐aortic lymph node metastasis; RV, Renal vein. *According to JSCCR classification.

Exploratory subgroup analysis for OS was performed in the PS‐matched cohort for each variate used to calculate PSs (Figure 4). In many subgroups, surgical resection had a favorable trend to improve survival compared to chemotherapy, especially in patients with poorly differentiated histology (HR [95% CI] 0.30 [0.11–0.83]), solitary clinical PALNM (0.33 [0.17–0.64]), or the presence of concurrent distant metastasis (0.34 [0.18–0.63]). Meanwhile, in patients with a primary tumor in the right colon (HR [95% CI] 0.81 [0.29–2.27]), multiple clinical PALNM (0.83 [0.48–1.43]), or the PALNM spread above RV (0.88 [0.32–2.40]), the survival impact of surgical resection for PALNM seemed to be small.

### 3.6 Sensitivity analysis

In addition, the sensitivity analysis was performed using a cohort excluding patients with missing detailed regimen data in the chemotherapy group. A total of 46 PS‐matched pairs were extracted from 225 patients and compared. The balance of selected variables, including age, sex, tumor location, histology, cT and cN classification, time to metastasis, the imaging finding of clinical PALNM, and concurrent distant metastasis, was well adjusted after matching.

Median follow‐up periods for survival in the PS‐matched cohort for sensitivity analysis were 3.49 (1.85–6.30) and 1.72 (1.29–2.64) years in the surgical resection group and in the chemotherapy group, respectively. Three‐ and 5‐year OS of patients in the chemotherapy group were 28.3% and 12.6%, respectively, with a MST of 1.73 years. Meanwhile, 3‐ and 5‐year OS of patients in the surgical resection group were 64.7% and 43.5%, respectively, with a MST of 4.37 years, which were significantly better compared to those in the chemotherapy group (Log‐rank *p* < 0.001).

## 4 DISCUSSION

The JSCCR‐PALNM project was the largest observational study for patients with PALNM from CRC conducted to date, and the present supplementary analysis sought to demonstrate the true impact of surgical intervention on survival for PALNM. After adjusting for patient background between the surgical resection and chemotherapy groups by PS matching, the survival of patients who underwent surgical resection and chemotherapy for PALNM were compared. Selected patients included 40.3% of those with distant metastasis other than PALNM, eventually resulting in a curative resection rate of 72.7% for those with surgical resection and a 5‐year RFS of 24.4% for those who achieved curative resection. Meanwhile, the MST of 4.37 years and 5‐year OS of 40.2% in the surgical resection group were significantly better compared to the MST of 2.49 years and 5‐year OS of 27.7% in the chemotherapy group.

The incidence of PALNM from CRC is relatively low, making research advances difficult in this field. Although several studies have suggested potentially better outcomes with surgical resection,^5^, ^7^, ^8^, ^9^, ^10^, ^11^, ^12^, ^13^, ^14^, ^15^, ^16^, ^17^, ^18^ the benefit of surgical resection on survival in these patients remains controversial. In 2010, Choi et al.^5^ compared patients who underwent surgical resection with those who did not, and found that surgical resection potentially improved survival. In 2021, Lee et al.^14^ reported promising results of PALN dissection on survival without any increase in postoperative complications. Furthermore, using a time‐varying covariate analysis, Kunitomo et al.^15^ reported on the clinical significance of surgical intervention for isolated PALNM as a multidisciplinary treatment strategy. Nevertheless, all of the aforementioned studies were retrospective observational studies with a small sample size, which may have introduced selection bias, leading to a false positive interpretation of the surgical intervention results.

The present analysis found that, although the curative resection rate and RFS still have room for improvement, surgical resection of PALNM significantly improved survival, as evidenced by an increase in MST of about 1.9 years and 5‐year OS of about 13% in the PS‐matched cohort. These results strongly suggest that surgical resection positively impacts the survival of patients with PALNM. Meanwhile, it would be premature to generalize the present results to all patients with PALNM from CRC. Although exploratory analysis, our subgroup analysis showed the heterogeneity of patients with PALNM. In patients with a primary tumor in the right colon, multiple clinical PALNM or the PALNM spread above RV, the survival impact of surgical resection for PALNM seemed to be small. These results also suggest that PALNM with these patients is inherently a part of systemic disease, and the management strategy should be mainly focused on systemic treatment.

The present study has some limitations. First, this is a supplementary analysis of the JSCCR‐PALNM project and was not pre‐planned. Thus, some critical variables were missing, the most important of which is detailed regimen data for the chemotherapy group. While the sensitivity analysis showed a similar trend to the primary analysis, the effect of an unknown chemotherapy regimen on the present results remains uncertain. Second, genetic data such as RAS, BRAF, MSI, and HER2 status were not included in CRFs, precluding any marker‐based discussions regarding indications for surgical intervention. Third, diagnostic criteria for PALNM differed between patients who underwent surgical resection and those who did not. Patients who underwent surgical resection were registered based on clinical and histological confirmation, while those who did not undergo surgical resection did not require histological confirmation, potentially leading to an overestimation of survival in the chemotherapy group. Fourth, the results may have been affected by unidentified non‐oncologic factors, such as performance status and comorbidities, which could not be adjusted for in the PS matching. Despite the above limitations, strengths of the present study include a cohort of patients who underwent surgical resection for PALNM which is the largest to date, the inclusion of a control group of patients without surgical resection during the same period in the same institutions, and adjustments based on PS matching. Significant improvement of OS in the surgical resection group after adjustment provides highly reliable evidence supporting the benefit of surgical intervention for PALNM.

## 5 CONCLUSIONS

In the present PS‐matched cohort, OS of patients who underwent surgical resection for PALNM was significantly better compared to those who did not. These results suggest that surgical intervention contributes to survival for patients with resectable PALNM.

## AUTHOR CONTRIBUTIONS

**Akira Ouchi:** Conceptualization; formal analysis; investigation; methodology; writing – original draft; writing – review and editing. **Koji Komori:** Supervision; writing – review and editing. **Sono Ito:** Data curation; project administration; writing – review and editing. **Yusuke Kinugasa:** Project administration; supervision; writing – review and editing. **Soichiro Ishihara:** Supervision; writing – review and editing. **Akio Shiomi:** Supervision; writing – review and editing. **Yukihide Kanemitsu:** Supervision; writing – review and editing. **Takeshi Suto:** Supervision; writing – review and editing. **Hiroki Takahashi:** Supervision; writing – review and editing. **Yoichi Ajioka:** Supervision; writing – review and editing.

## CONFLICT OF INTEREST STATEMENT

YK (Yusuke Kinugasa) is an editorial member of *Annals of Gastroenterological Surgery*.

## ETHICS STATEMENT

Approval of the research protocol: The protocol review committee of the JSCCR (95–1) and independent ethics committees of the Aichi Cancer Center Hospital (IR031044) and other participating institutions approved the present supplementary analysis.

Informed consent: Informed consent was obtained by an opt‐out method from all patients participating in this study.

Registry and the registration no. of the study/trial: N/A.

Animal studies: N/A.
